# Interplay between nutrition, microbiota, and immunity in rotavirus infection: insights from human and animal models

**DOI:** 10.3389/fvets.2025.1680448

**Published:** 2025-09-08

**Authors:** Wenying Huo, Yingying Qiao, Enkai Li, Mengyun Li, Long Che

**Affiliations:** ^1^College of Animal Science and Technology, Henan University of Animal Husbandry and Economy, Zhengzhou, China; ^2^Department of Molecular Microbiology, Washington University School of Medicine, St. Louis, MO, United States

**Keywords:** nutrition, microbiota, immunity, RV, gut health

## Abstract

Rotavirus (RV) remains a leading cause of severe gastroenteritis in infants and young animals worldwide, contributing to significant morbidity and mortality despite the availability of vaccines. The gastrointestinal tract’s health, shaped by complex interactions between nutrition, the gut microbiota, and the host immune system, plays a crucial role in RV pathogenesis and outcomes. Emerging evidence suggests that dietary components not only influence the composition and function of the gut microbiota but also modulate immune responses essential for protection against RV. This review integrates findings from human and animal models to elucidate the interplay between nutrition, microbiota, and immunity in the context of RV infection. We aim to shed light on the mechanisms by which dietary factors and microbial communities influence RV susceptibility and severity, and how this knowledge could guide the development of more effective preventive and therapeutic strategies.

## 1 Introduction

Rotavirus (RV) remains one of the leading causes of severe gastroenteritis, particularly among infants and young children and animals, causing substantial global morbidity and mortality. Despite advances in vaccination, RV infections continue to present significant public health challenges, especially in resource-limited regions (1). A growing body of research has underscored the pivotal role of gut health in modulating the immune response to viral infections. The gut microbiota, a complex community of microorganisms residing in the intestines, plays a crucial role in both maintaining intestinal homeostasis and modulating immune functions. Dysbiosis, or imbalance in the microbiota, has been implicated in exacerbating disease outcomes during RV infections (2). The gut microbiota plays a central role in shaping immune function, and nutrition—an important determinant of immune responses—further modulates these interactions, influencing susceptibility to RV infection. Dietary components can alter the composition and function of the gut microbiota, thereby impacting the host’s immune response to infections (3). Recent studies leveraging human and animal models have highlighted potential therapeutic avenues through nutritional and microbiota-based interventions, offering potential avenues for therapeutic strategies (4). This review aims to explore the intricate interplay between nutrition, gut microbiota, and immunity in the context of RV infection.

## 2 RV biology and immune response

### 2.1 Host immune responses to RV

RV is a non-enveloped, double-stranded RNA virus belonging to the Reoviridae family. It is characterized by an 11-segmented genome encapsulated within a triple-layered protein capsid, which is essential for its stability and infectivity (5). RV infection triggers innate immunity at the intestinal epithelium. Enterocytes detect viral dsRNA via RIG-I, MDA5, and TLR3, which activate IRF3 and NF-κB, driving type I (IFN-*α*/*β*) and type III (IFN-*λ*) interferon production (6). These interferons establish an antiviral state by inducing interferon-stimulated genes (ISGs) that limit viral replication. Additionally, RV modulates host responses through viral proteins such as NSP1, which antagonizes IFN signaling by targeting IRF3 and other regulatory factors for proteasomal degradation (6, 7). In the adaptive phase, mucosal immunity is critical for protection. The infection stimulates gut-associated lymphoid tissue (GALT), particularly Peyer’s patches and mesenteric lymph nodes, where antigen-presenting cells such as dendritic cells activate naïve CD4^+^ T cells, leading to B cell class switching and the generation of IgA-producing plasma cells (8). These plasma cells home to the intestinal lamina propria via CCR9 and α4β7 integrins and secrete virus-specific secretory IgA (sIgA) that neutralizes virus particles in the lumen and on the epithelial surface, preventing reinfection. CD8^+^ cytotoxic T lymphocytes also play a role in clearing infected cells, especially in the early stages of infection (8). Protective immunity is largely non-sterilizing but is enhanced by repeated exposure, which boosts both the magnitude and breadth of sIgA and systemic IgG responses (5). In vaccine contexts, the generation of intestinal sIgA and memory B cells has been correlated with long-term protection, though no absolute immunologic correlate of protection has been universally accepted (9).

### 2.2 Limitations of RV vaccines

Recent data further underscore the geographic disparities in rotavirus vaccine performance. According to WHO surveillance and Gavi reports, vaccine effectiveness exceeds 80–90% in many high-income countries but drops significantly in low- and middle-income countries (LMICs), often ranging from 50 to 60% (10). The limitations of RV vaccines in providing effective immune protection are multifaceted, involving both host and viral factors that compromise efficacy, particularly in LMICs. A major constraint is the incomplete understanding of immune correlates of protection; while serum IgA and neutralizing antibodies have been associated with protection, they are not fully reliable predictors of vaccine efficacy, complicating the assessment and improvement of vaccines (11). The mucosal immunity required for robust protection is often inadequately induced, particularly in settings where environmental factors like malnutrition, high pathogen exposure, and environmental enteric dysfunction impair immune responses (12, 13). Maternal antibodies transferred via the placenta or breast milk can neutralize vaccine virus replication in the infant gut, dampening the immune response (12). Additionally, the gut microbiome composition, altered by geography and nutrition, influences vaccine take and efficacy, with evidence suggesting that dysbiosis can reduce immunogenicity (13).

The genetic diversity of RV also presents a significant hurdle; although current vaccines provide some cross-protection, they are primarily based on specific strains, and the continual emergence of novel genotypes may limit broad protective coverage (1, 14). Moreover, vaccine-induced immunity may wane after the first 2 years of life, particularly in LMICs, leaving children susceptible to later infections (13). There are operational challenges as well, including the need for cold chain logistics and multiple doses, which can limit vaccine coverage and timely administration (14). These combined factors highlight the need for next-generation vaccines that can induce stronger and more durable mucosal immunity, broaden strain coverage, and overcome host and environmental barriers to ensure equitable global protection against RV (11, 14).

## 3 Gut microbiota and RV infection

### 3.1 Influence on susceptibility and severity

The gut microbiota profoundly influences host susceptibility, immune responses, and vaccine efficacy against RV through modulation of intestinal barrier function, immune signaling, and viral replication dynamics. Heyman et al. (15) found that germ-free mice infected with RV exhibited increased intestinal permeability and abnormal absorption of macromolecules like bovine serum albumin, suggesting that microbiota are critical in maintaining tight junction integrity and limiting paracellular leakage during infection. Hulst et al. (16) provided molecular evidence from germ-free piglets, where RV infection triggered an exaggerated transcriptional response in the jejunum, particularly upregulation of genes related to innate immunity, such as interferon-stimulated genes (ISGs), acute phase reactants, and inflammatory cytokines, indicating that microbiota normally calibrate the epithelial and mucosal immune tone to prevent hyperactivation. Uchiyama et al. (17) further demonstrated that broad-spectrum antibiotic treatment, which depletes gut microbiota, significantly reduced RV replication in mice and simultaneously enhanced virus-specific humoral immunity, including higher titers of IgA and IgG. This suggests that certain bacterial species may provide metabolites or immune cues that favor viral replication while dampening adaptive immune responses. Ngo et al. (18) extended these findings by identifying microbial taxa, particularly some Clostridia and Bacteroidetes members, that impair RV vaccine efficacy by suppressing type I interferon pathways and antigen-presenting cell functions, thereby reducing the priming of protective immunity. Collectively, these studies underscore that the microbiota shapes the epithelial barrier, regulates innate immune thresholds, modulates viral replication niches, and influences both infection outcomes and vaccine responses to RV.

### 3.2 Microbial metabolites and immunomodulation

The gut microbiota produces a diverse array of metabolites that profoundly influence the host’s immune system, antiviral activity, and barrier function (19–21). Several microbial metabolites and compounds modulate RV infection through diverse cellular pathways and antiviral mechanisms (Table 1). Butyrate, a short-chain fatty acid produced by gut bacteria, exerts protective effects against RV-induced damage by attenuating endoplasmic reticulum (ER) stress-mediated apoptosis via suppression of the PERK-eIF2α signaling pathway, thereby reducing epithelial cell death (22). Additionally, sodium butyrate preserves intestinal barrier integrity by activating the AMPK-Nrf2 signaling pathway, leading to enhanced antioxidant defenses and tight junction protein expression in IPEC-J2 cells (23–25).

**Table 1:** Bioactive compounds and host-derived factors modulating RV infection: mechanisms and outcomes across models.

Compound/factor	Model/system	Mechanism of action	Key outcomes	Reference
Sodium butyrate	IPEC-J2 (porcine IECs)	Inhibits ER stress-mediated apoptosis via PERK-eIF2α signaling pathway	Protects cells from RV-induced apoptosis	(22)
Sodium butyrate	IPEC-J2	Activates AMPK-Nrf2 signaling pathway to protect intestinal epithelial barrier	Preserves barrier integrity during RV infection	(23)
Bile acids (e.g., chenodeoxycholic acid, deoxycholic acid)	Cell cultures	Inhibit RV replication; FXR agonists block viral replication through unknown precise mechanisms	Reduced viral replication	(26)
Bile acids	General/Animal Studies	Modulate enteric virus replication via bile acid receptor-mediated pathways (FXR, TGR5)	Can suppress or enhance RV infection depending on type	(27)
Serotonin	In vivo/Pigs/Mice	Cross-talk with RV promotes diarrhea via modulation of ENS and intestinal secretion	Exacerbates diarrheal symptoms	(29)
Bacterial-derived sialidases	Porcine RV OSU in vitro	Inhibits RV infection by interfering with virus attachment and entry (early infection steps)	Reduced viral replication	(28)

Beyond butyrate, bile acids and synthetic farnesoid X receptor (FXR) agonists inhibit RV replication by disrupting viral transcription and replication processes, suggesting bile acid receptors play a regulatory role in antiviral defenses (26, 27). Bacterial-derived sialidases impede porcine RV replication by cleaving host cell surface sialic acids, interfering with viral attachment and entry during early infection stages (28).

Additionally, the interaction between RV and the host’s serotonergic system influences diarrheal outcomes, with RV triggering serotonin release that modulates gut motility and secretion, exacerbating diarrhea (29). Metabolomic studies further highlight that host–microbe interactions shape the metabolic landscape influencing RV pathogenesis, though specific metabolic mediators require further elucidation (30). Lastly, carbohydrate malabsorption syndrome contributes to the pathogenesis of RV diarrhea by promoting osmotic imbalances and altering the gut microbiota composition, thereby aggravating disease severity (31). Together, these findings emphasize the therapeutic potential of microbial metabolites like butyrate, bile acids, and bacterial enzymes in mitigating RV infection and its associated intestinal damage.

### 3.3 Microbiota and vaccine responses

The gut microbiota plays a significant role in shaping both mucosal and systemic immune responses to vaccines, including those against RV (32). Multiple bacterial species modulate RV (RV) infection through distinct mechanisms involving direct viral inhibition, microbiota modulation, immune priming, and glycan interactions (Table 2). *Lactobacillus rhamnosus* GG and *Lactobacillus acidophilus* reduce RV diarrhea severity by enhancing mucosal barrier integrity, producing antiviral metabolites, and modulating immune responses such as increasing sIgA levels (33–35). *Bifidobacterium longum* inhibits RV replication *in vitro* by competitive exclusion and modulation of host cell antiviral pathways, such as upregulating interferon-stimulated genes (36).

**Table 2:** Effects of bacterial species on RV infection and vaccine response in human and animal models.

Bacterial species	Model/system	Effect on RV infection	Mechanism/key findings	Reference
*Lactobacillus rhamnosus* GG	Human clinical, in vitro	Reduced duration of diarrhea; prophylactic effect	Enhances immune response, potential direct interaction with virus	(33, 35)
*Lactobacillus acidophilus*	In vitro, pediatric clinical	Inhibits RV infection; reduces diarrhea	Modulation of intestinal environment, antiviral activity	(34)
*Bifidobacterium longum*	Caco-2 cells, neonatal mouse	Inhibits RV infection; reduces diarrhea severity	Enhances mucosal immunity, direct inhibition	(36)
Segmented Filamentous Bacteria (SFB)	Mouse	Prevents and cures RV infection	Enhances Th17 and retinoic acid receptor-mediated signaling	(37, 38)
*Lactobacillus reuteri*	Pig feces, porcine RV model	Inhibits porcine RV infection	Antimicrobial activity, competitive exclusion	(40)
*Lactobacillus casei*	Human intestinal cells	Inhibits RV infection in vitro	Secretes glycan-modifying soluble factors	(39)
*Bacteroides thetaiotaomicron*	Human intestinal cells	Inhibits RV infection	Produces glycan-modifying metabolites	(39)
*Bifidobacterium bifidum* and *Streptococcus thermophilus*	Hospitalized infants	Prevents diarrhea and RV shedding	Competitive exclusion, gut environment modulation	(47)
*Lactobacillus ruminis*	Caco-2, neonatal mouse	Reduces RV infection	Antiviral metabolites, immune modulation	(36)
Various lactic acid bacteria	Human infant mucus samples	Enhanced adherence during RV infection	Improved colonization during infection	(41)
Unclassified glycan-degrading bacteria	In vitro, animal models	Inhibits RV infection	Glycan-mediated virus-bacteria-host interactions	(44)
LPS-rich bacteria (e.g., Enterobacteriaceae)	Human infants (South Africa)	Associated with reduced RV vaccine shedding	Modulates TLR4 and IL-8 expression	(43)
Lactic acid bacteria (multiple species)	Gnotobiotic pigs	Influence immune cell distribution during RV infection	Modulate monocytes/macrophages and dendritic cells	(42)
Probiotic bacteria mix	IPEC-J2 cells	Modulates porcine RV infection	Immunomodulation, cell viability effects	(116)

Segmented filamentous bacteria (SFB) exhibit potent anti-RV effects by inducing a Th17-mediated immune response and enhancing retinoic acid receptor signaling, which primes intestinal epithelial cells for rapid interferon production, creating an antiviral state that blocks RV replication (37, 38). Similarly, *Bacteroides thetaiotaomicron* and *Lactobacillus casei* secrete glycan-modifying enzymes and soluble factors that alter host cell surface glycans, thereby impeding RV binding to its receptors, specifically by reducing sialic acid-containing glycan availability (39). *Lactobacillus reuteri*, isolated from pigs, exhibits bile tolerance and produces antimicrobial compounds like reuterin that inhibit both RV and co-infecting bacterial pathogens, indirectly reducing RV infectivity by controlling microbial dysbiosis (40). Probiotics also enhance mucus production and fortify tight junction proteins, limiting RV penetration (41). Furthermore, probiotic colonization in neonatal gnotobiotic pigs modulates dendritic cell and macrophage populations, promoting antiviral cytokine production such as IL-12 and IFN-*γ* (42). Lipopolysaccharide (LPS)-rich bacteria increase TLR4 activation, influencing innate immune responses that modulate live attenuated RV vaccine virus shedding (43). Glycan-mediated interactions between certain commensals and RV can competitively block viral entry by saturating glycan receptors on epithelial cells, adding an additional antiviral defense layer (44).

RV infection itself reshapes the gut microbiome by increasing glycan availability in the ileum, facilitating the overgrowth of specific bacteria that may enhance or inhibit subsequent RV infection (45). Human studies demonstrate that antibiotic-mediated microbiome depletion reduces RV vaccine immunogenicity by impairing microbial-derived adjuvant signals necessary for optimal immune priming (32). Specific microbiota profiles correlate with oral RV vaccine efficacy, particularly involving bacteria that modulate bile acid metabolism and TLR signaling (2, 18, 46). Early probiotic supplementation with *Bifidobacterium bifidum* and *Streptococcus thermophilus* reduced RV diarrhea and viral shedding, potentially via enhanced colonization resistance and modulation of gut pH to inhibit RV replication (47). Overall, these findings underscore that diverse bacterial taxa employ multi-modal mechanisms — including modulation of host immunity, competitive exclusion, glycan receptor modification, and metabolic interactions — to mitigate RV infection and improve vaccine responses.

## 4 Nutritional factors modulating RV infection and immunity

### 4.1 Macronutrients

Adequate intake of macronutrients and micronutrients is fundamental to maintaining a competent immune system and mitigating the impact of RV infection (48). Protein-energy malnutrition (PEM) profoundly disrupts intestinal integrity and immune defense against RV (RV) infection through multiple mechanisms (Table 3). In neonatal pigs and mice, PEM impairs small intestinal epithelial regeneration and mucosal repair post-RV infection by reducing villus height, crypt depth, and epithelial cell proliferation, thereby delaying recovery (49–51). Mechanistically, protein deficiency downregulates tight junction proteins and mucins, weakening the epithelial barrier and facilitating viral invasion (3). PEM also diminishes expression of pattern recognition receptors like TLR3 and RIG-I, essential for recognizing RV and initiating antiviral responses (48). Concurrently, protein malnutrition skews immune responses by reducing type I and III interferons, IL-22, and antimicrobial peptides, all critical for limiting RV replication (3). Furthermore, PEM alters gut microbiota composition—favoring pathobionts and depleting beneficial commensals—which exacerbates inflammation and diminishes the efficacy of oral RV vaccines (52, 53).

**Table 3:** Role of protein, energy, fatty acids, and carbohydrates in RV infection and immunity.

Nutrient	Study model	Key findings	Mechanisms/Pathways	References
Protein/protein-energy	Neonatal pigs (PEM)	PEM delays small intestinal repair post-RV infection; worsens villus atrophy, reduces crypt proliferation	Impaired epithelial regeneration; reduced tight junction proteins; suppressed TLR3, RIG-I expression; diminished type I/III IFN, IL-22	(3, 49, 50)
	Mice	PEM alters IgA responses to RV vaccine but vaccine efficacy remains	Reduced mucosal IgA, altered adaptive immunity	(122)
	Gnotobiotic pigs	Protein deficiency reduces efficacy of oral RV vaccine; impacts gut microbiota	Microbiota dysbiosis reduces vaccine-induced protection	(53, 123)
	Pigs, Mice	Protein malnutrition alters gut microbiota composition during RV infection	Loss of beneficial bacteria, overgrowth of pathobionts	(52)
	Pigs	Protein malnutrition alters ACE2, tryptophan metabolism, adaptive immune responses	Dysregulated tryptophan-kynurenine pathway; weakened mucosal immunity	(48)
	Suckling rats & mice	Whey protein concentrate ameliorates RV diarrhea; improves immune response	Increases sIgA, anti-inflammatory cytokines; enhances mucosal immunity	(54, 56)
	Neonatal pigs	Animal plasma protein reduces intestinal damage during RV infection	Promotes intestinal repair, enhances anti-inflammatory responses	(55)
	*In vitro*	Whey protein lipid concentrate inhibits RV replication in porcine and human cells	Blocks viral attachment and entry via milk fat globule membrane components	(59)
	Mice	Undernutrition weakens immune response to RV	Reduced mucosal immunity, impaired barrier function	(124)
Fatty acids	In vitro (cell culture)	Saturated fatty acids (palmitic/stearic) enhance RV infectivity	Promotes viral entry/uncoating by altering membrane fluidity	(60)
	Cell culture, Lipidomics	Ceramide metabolism supports RV replication	Ceramide-rich domains necessary for viral assembly and budding	(61)
Carbohydrates	In vitro	Carbohydrates aid correct folding of VP7 via disulfide bond formation	Essential for mature, infectious virion assembly	(62)
	Human infants	Carbohydrate malabsorption in RV diarrhea worsens osmotic diarrhea	Unabsorbed carbs increase luminal osmolality, worsening diarrhea	(63)
	In vitro	Glycolipids and sialic acid residues act as RV receptors	Facilitates viral attachment and entry	(64, 65)
	Piglets	POP reduces diarrhea, viral load, and inflammation in porcine RV-infected piglets	Enhances mucosal barrier integrity; reduces pro-inflammatory cytokines (IL-6, TNF-*α*); boosts antioxidant capacity	(67)
	In vitro	POP inhibits porcine RV replication in IPEC-J2 cells	Directly reduces viral replication; modulates NF-κB pathway to suppress inflammation	(66)

Dietary supplementation with whey protein concentrates or animal plasma enhances intestinal repair by increasing anti-inflammatory cytokines (e.g., IL-10) and sIgA, and restoring mucosal immunity, thereby reducing RV-associated diarrhea and intestinal damage (54–58). Milk fat globule membrane components specifically block RV replication by disrupting viral attachment and entry processes (59). In terms of energy and fatty acids, saturated fatty acids such as palmitic and stearic acids enhance RV infectivity by promoting membrane fluidity and facilitating viral entry and uncoating (60). In contrast, oleic acid supplementation and modulation of lipid droplet metabolism can reduce RV replication by altering host lipid metabolism, including beta-oxidation and lipid droplet formation, which RV exploits for its replication complexes (61). Ceramide metabolism is particularly critical, as ceramide-enriched membrane domains are necessary for RV assembly and budding; disruption of ceramide synthesis impairs these viral processes (61).

Carbohydrates impact RV infection both structurally and functionally. Proper glycosylation and disulfide bond formation in RV proteins, particularly VP7, are carbohydrate-dependent processes necessary for forming mature, infectious virions (62). Malabsorption of carbohydrates during RV infection leads to osmotic diarrhea, exacerbating fluid loss and disease severity (63). Intestinal glycolipids and glycoconjugates rich in sialic acid residues act as critical cell surface receptors for RV, mediating viral attachment and entry (64, 65). Inhibitory glycans and synthetic carbohydrate analogs can competitively block these binding sites, preventing infection (62). Additionally, *Portulaca oleracea* L. polysaccharide (POP) has been shown to inhibit porcine RV both *in vitro* and *in vivo* by reducing viral replication, enhancing mucosal barrier function, modulating the NF-κB pathway, and lowering inflammatory cytokines such as IL-6 and TNF-*α* (66, 67). Altogether, adequate intake of protein, energy, specific fatty acids, and digestible carbohydrates not only supports epithelial and immune defenses but also limits RV replication and pathogenesis by directly modulating viral entry, replication, and host–microbe interactions.

### 4.2 Micronutrients

Micronutrients are equally vital. Vitamin A and D have been extensively studied for their modulatory effects on RV (RV) infection and immunity (Table 4). Vitamin A deficiency (VAD) exacerbates RV infection through compromised gut integrity and impaired immune responses. In mice, VAD led to severe diarrhea, villous atrophy, and extensive gut involvement, reducing epithelial regeneration and delaying viral clearance (68, 69). Mechanistically, VAD diminished mucosal and systemic immunity by decreasing RV-specific intestinal IgA and systemic IgG, as well as impairing T cell proliferation (70). In gnotobiotic piglets, VAD prenatally acquired from sows resulted in reduced maturation of dendritic cells and suppressed type I interferon responses (IFN-*α*, IL-12), weakening innate defenses (71). Adaptive immunity was also compromised, with reduced B cell antibody responses (IgA, IgG) and impaired CD4 + and CD8 + T cell responses to both vaccination and RV challenge (72, 73).

**Table 4:** Effects of vitamin A and vitamin D on RV infection: summary of experimental and clinical findings across animal and human models.

Vitamin	Study model	Intervention	Key Findings	Mechanisms	Reference
Vitamin A	Mouse	Vitamin A Deficiency (VAD)	VAD worsens RV infection and gut pathology	Impaired mucosal integrity and immune responses	(124)
	Mouse	VAD	Reduced immune responses (IgA, IFN-*γ*) against EDIM RV	Compromised mucosal and systemic immunity	(70)
	Mouse (CD-1)	VAD	Aggravated RV infection with severe gut damage	Villous atrophy, crypt hyperplasia	(69)
	Gnotobiotic Piglets	Prenatal VAD	Impaired innate (IFN-α, MxA) and adaptive responses to human RV	Suppressed NK cell, DC function	(71)
	Gnotobiotic Piglets	Prenatal VAD	Reduced B/T cell responses to monovalent HRV vaccine and challenge	Diminished virus-specific IgA/IgG, T-cell proliferation	(72)
	Gnotobiotic Piglets	Prenatal VAD	Reduced adaptive immune responses to RotaTeq® vaccine	Poor vaccine-induced protection	(73)
	Pregnant Sows & Piglets	Oral vitamin A supplementation in VAD sows	Enhanced maternal immunity, higher piglet passive protection	Boosted maternal antibody titers	(74)
	Sows	VAD + supplementation	VAD dampened T cell & innate immunity, supplementation improved it	Enhanced NK cells, IFN-*γ*, CD8 + T cells	(75)
Vitamin D	Piglets & IPEC-J2 Cells	Vitamin D3 supplementation	Alleviated RV infection, reduced viral load	Enhanced autophagy via mTOR and LC3 pathways	(76)
	Piglets	Dietary vitamin D	Reduced immune overactivation post-RV	Regulated RIG-I, MDA5, and IRF3 signaling	(81)
	Piglets & IPEC-J2 Cells	25-Hydroxyvitamin D3 treatment	Inhibited RV replication	Upregulated RIG-I, IRF7 expression	(78)
	IPEC-J2 Cells	1α,25-hydroxyvitamin D3	Protected against RV-induced ferroptosis	Modulated ATF3-SLC7A11-GPX4 axis	(80)
	Humans (Children with RV Diarrhea)	Serum vitamin D measured	Low vitamin D associated with higher risk/severity of rotaviral diarrhea	Correlation, not intervention	(77)
	Mice & Cells	Vitamin D3 treatment	Reduced RV infection via miR-155-5p regulation of TBK1/IRF3	Modulated innate antiviral signaling	(79)

Vitamin A supplementation effectively reversed these deficits. Oral vitamin A in VAD pregnant sows enhanced RV-specific IgA and IgG in colostrum and serum, strengthening maternal adaptive immunity and passive protection in piglets (74). Further, supplementation restored innate immune function by increasing natural killer (NK) cell activity and IFN-*γ* production, and promoted robust CD8 + T cell responses (75). Clinically, vitamin A supplementation improved outcomes in preschool children with acute RV diarrhea, shortening disease duration and severity. Mechanistically, vitamin A and its metabolites (retinoids) enhance antiviral defenses by supporting mucosal immunity, promoting epithelial repair, and modulating the Th1/Th2 cytokine balance (73).

Vitamin D supplementation similarly alleviates RV infection through distinct pathways. In pigs and IPEC-J2 cells, vitamin D3 activated autophagy by upregulating Beclin1 and LC3, promoting viral degradation and reducing replication (76). In humans, low serum vitamin D was associated with higher susceptibility to rotaviral diarrhea in children (77). Mechanistically, 25-hydroxyvitamin D3 inhibited RV replication by suppressing the RIG-I and MDA5 pathways, leading to lower IFN-*β* production and inflammatory signaling in infected IPEC-J2 cells (78). Vitamin D also regulates miR-155-5p. This modulation of the TBK1/IRF3 axis tempers excessive interferon responses while maintaining control of viral replication (79). Additionally, 1α,25-dihydroxyvitamin D3 protected against RV-induced ferroptosis in intestinal epithelial cells via the ATF3-SLC7A11-GPX4 pathway, enhancing cellular antioxidant defenses and survival (80). In pigs, dietary vitamin D also modulated RIG-I signaling and reduced pro-inflammatory cytokine expression during RV infection, contributing to a balanced immune response (81). Collectively, these findings underscore that vitamin A primarily bolsters gut barrier function, mucosal IgA, and T cell immunity, whereas vitamin D mitigates RV pathogenesis via autophagy activation, innate immune signaling modulation, miRNA-mediated regulation, and protection against infection-induced ferroptosis, all contributing to improved viral control and vaccine efficacy.

Zinc exerts multifaceted protective effects against RV infection through both immunological and direct antiviral mechanisms. Clinically, higher serum zinc concentrations are associated with a lower incidence of RV gastroenteritis, partly by enhancing the mucosal immune response, particularly in infants receiving delayed RV vaccination (82). Zinc supplementation reduces the duration and severity of diarrhea by promoting epithelial barrier integrity, reducing intestinal permeability, and enhancing mucosal repair (83). Immunologically, zinc upregulates immune globulins (IgA, IgG), complement proteins, and suppresses pro-inflammatory cytokines including TNF-*α*, IL-6, and IL-17, which are typically elevated during RV infection-induced intestinal inflammation. Moreover, zinc influences gut microbiota composition, facilitating microbial shifts that support mucosal health and recovery post-infection (84). Mechanistically, zinc ions play a critical structural role in the RV virion: they stabilize the VP6 protein, a major capsid component essential for viral assembly and maintenance of the triple-layered particle, directly impacting viral integrity and infectivity (85). Additionally, zinc oxide nanoparticles have been shown to directly inhibit RV replication *in vitro*, likely through the generation of reactive oxygen species (ROS) that damage viral components and disrupt replication cycles (86). The economic benefits of zinc supplementation in RV management, particularly in reducing treatment duration and healthcare costs, further emphasize its therapeutic potential. Combination therapies with zinc, selenium, and probiotics demonstrate synergistic benefits by collectively enhancing immune resilience and antiviral defenses (87).

Copper exhibits antiviral properties against RV, with *in vitro* evidence showing that copper oxide nanoparticles suppress RV multiplication. This effect is mechanistically linked to ROS generation, which leads to oxidative stress and denaturation of viral proteins, impairing the viral life cycle (88).

Calcium plays a dual role in the RV replication cycle. Physiologically, calcium ions facilitate the early stages of RV infection by mediating virus attachment and entry into host cells through endocytic pathways (89). However, intracellular calcium depletion alters viral protein processing: chelation of calcium activates the RV RNA-dependent RNA polymerase (RdRp), enhancing viral transcription (90). Conversely, sustained calcium depletion impairs the oligomerization of viral proteins within the endoplasmic reticulum, blocking proper virus maturation and assembly (91). Thus, calcium homeostasis is critical for balancing viral replication, assembly, and maturation processes. Collectively, these findings highlight the multifaceted roles of minerals in modulating RV infection and replication across clinical and molecular levels (Table 5).

**Table 5:** Summary of the effects and mechanisms of zinc, copper and calcium on RV infection.

Mineral	Study model	Intervention/exposure	Key findings	Mechanism/outcomes	Reference
Zinc	Human (Infants)	Serum zinc levels + delayed RV vaccine	Higher serum zinc reduced risk of RV gastroenteritis	Enhanced mucosal immunity and vaccine response	(82)
	Human (Infants)	Probiotics + zinc with RV vaccine	No significant effect on vaccine immune response	Zinc alone insufficient to boost immunogenicity	(125)
	Human (Children)	Probiotics + zinc + lactose-free formula	Reduced duration of RV diarrhea	Zinc aids epithelial repair and immunity	(83)
	Human (Children)	Zinc + conventional therapy	Improved gut microbiota recovery	Modulation of microbiome composition	(84)
	In vitro	Zinc ion presence	Controls VP6 capsid assembly/stability	Zinc essential for viral structural integrity	(85)
	In vitro	Zinc oxide nanoparticles	Inhibited RV and MDR *A. baumannii* growth	Antiviral via ROS production	(86)
	Human (Children)	Probiotics + zinc + selenium	Improved clinical outcomes	Synergistic immune enhancement	(87)
Copper	In vitro	Copper oxide nanoparticles	Inhibited RV replication	Direct antiviral action via ROS	(88)
Calcium	In vitro	Calcium modulation	Essential for viral entry	Facilitates virus-cell binding	(89)
	In vitro	Calcium chelation	Activated RV RNA polymerase	Calcium depletion activates transcription	(90)
	In vitro	Calcium depletion	Blocked RV maturation	Disrupts protein folding, assembly in ER	(91)

### 4.3 Human milk components

Human milk contains multiple bioactive components that protect against RV (RV) infection through distinct and synergistic mechanisms (Table 6). Human milk oligosaccharides (HMOs) inhibit RV infection by acting as soluble decoy receptors that mimic host cell surface glycans, particularly histo-blood group antigens (HBGAs), which are recognized by the viral VP8* protein, thereby preventing RV attachment and entry into enterocytes (92, 93). In piglet models, HMOs not only reduce diarrhea severity and viral shedding but also enhance mucosal immunity by increasing intestinal sIgA and modulating cytokine responses, while reshaping the colonic microbiota toward protective bacterial profiles such as increased Bifidobacteria (94, 95). The milk microbiome itself, in concert with HMOs, promotes the colonization of beneficial microbes that can enhance gut barrier function and indirectly reduce RV infection susceptibility (95).

**Table 6:** Protective effects of human milk components against RV infection: mechanisms and experimental evidence.

Human milk component	Experimental model	Mechanism of action	Key findings	Reference
HMOs	In vitro (MA104 cells), piglets	Act as decoy receptors, blocking virus binding to intestinal cells.	Inhibited infectivity of human RV in vitro; reduced diarrhea and viral shedding in piglets; modulated mucosal immunity and gut microbiota.	(92–95)
Mucin (MUC1, MUC4)	In vitro, animal models	Inhibits RV replication by blocking virus-cell interactions via glycan-mediated binding interference.	Human milk mucin prevents RV replication and gastroenteritis in vitro and *in vivo* models.	(96)
Lactadherin	Human cohort, in vitro	Binds directly to RV particles, preventing attachment to epithelial cells.	Presence of lactadherin in human milk correlates with reduced symptomatic RV infection in breastfed infants.	(97)
Extracellular vesicles (EVs)	In vitro (Caco-2, MA104 cells)	Contain bioactive molecules that inhibit viral entry and replication.	Human colostrum-derived EVs prevented infection by human RV and respiratory syncytial virus in vitro.	(98)
Anti-RV antibodies (IgA, IgG)	Human milk samples	Neutralization of virus directly through antibody binding, preventing infection and reducing viral replication.	Quantified anti-RV antibodies with confirmed neutralizing activity; higher levels associated with protection.	(99)
Milk proteins (Lactoferrin, etc.)	In vitro (MA104 cells)	Various bioactive proteins inhibit virus by direct binding or enhancing immune defense.	Both human and bovine milk proteins showed inhibitory effects on RV infectivity.	(101)
Human milk microbiome and HMOs	Human infants, gnotobiotic pigs	Synergistically modulate the infant gut microbiome, enhancing innate immunity and resistance to RV infection.	Breastmilk microbiome and HMOs shaped the infant gut microbiome, reducing susceptibility to neonatal RV infection.	(95)

Mucins (MUC1, MUC4) present in human milk exhibit sialylated and fucosylated glycans that bind RV particles, physically blocking the interaction with epithelial cell receptors necessary for viral internalization (96). Lactadherin, another glycoprotein, contains phosphatidylserine-binding domains that enable it to bind directly to RV outer capsid proteins, interfering with viral attachment and entry into enterocytes; higher lactadherin concentrations in milk are associated with reduced symptomatic RV infections in infants (97).

Extracellular vesicles (EVs) isolated from colostrum carry miRNAs, lipids, and proteins capable of modulating host cell antiviral responses, including blocking viral entry, possibly through receptor competition or modulation of intracellular signaling pathways involved in viral replication (98). Anti-RV antibodies (IgA, IgG) in milk directly neutralize RV by binding to viral capsid proteins, preventing attachment, facilitating agglutination, and promoting immune exclusion in the gut lumen (99).

Additionally, novel glycans in human milk have been identified using glycan microarrays; these glycans, particularly those with unique sialylation and fucosylation patterns, act as decoy receptors targeting specific RV strains prevalent in neonates (100). Milk proteins, such as lactoferrin, inhibit RV by binding to viral particles or blocking cell surface receptors, while also possessing iron-chelating properties that can impair viral replication (101). Lastly, studies on whole breastmilk show that certain components can inhibit the replication of live oral RV vaccines *in vitro*, indicating that while protective, some milk constituents may impede vaccine virus replication, potentially affecting seroconversion rates in infants (102). Collectively, these human milk factors provide both direct antiviral effects and indirect immunomodulatory actions that contribute to the protection of infants against RV infection.

### 4.4 Dietary fibers, prebiotics, and probiotics

Dietary fibers, prebiotics, and probiotics represent nutritional strategies with significant potential to modulate the gut microbiota, enhance immune responses, and confer protection against RV infection (Table 7) (103–105). Dietary fiber, particularly rice bran, protects against RV (RV) infection by multiple mechanisms, including enhancing intestinal barrier integrity, modulating gut microbiota, and promoting Th1-biased immune responses. In gnotobiotic pigs, rice bran supplementation reduced RV diarrhea and elevated IFN-*γ* and IL-12 production, boosting the response to RV vaccination (106). Rice bran also increased the abundance of Lactobacillus and Bifidobacterium, improved mucin production, and upregulated innate immune genes such as TLRs and antimicrobial peptides, leading to stronger epithelial defenses (104, 107). Prebiotics, such as the scGOS/lcFOS mixture, decreased RV-induced diarrhea in suckling rats by enriching Bifidobacterium and Lactobacillus populations, which enhance SCFA production, mucosal IgA secretion, and the expression of tight junction proteins, maintaining gut permeability (108–110). Prebiotic supplementation, together with bifidobacteria, enhanced both systemic and mucosal immunity in mice, with increased RV-specific IgA, IgG responses, and higher levels of IL-4 and IFN-*γ*, indicating balanced Th1/Th2 responses (111). Similarly, a prebiotic-postbiotic mixture strengthened gut barrier function, reduced intestinal permeability, and modulated inflammatory cytokines such as IL-10 and TNF-*α* in a neonatal rat RV infection model (112). Probiotics, including *Bifidobacterium breve*, *Lactobacillus rhamnosus* GG, and *Bacillus clausii*, have shown strain-specific antiviral effects through competitive exclusion, production of antiviral metabolites, and modulation of host immunity. For instance, *B. breve* M-16 V prevented diarrhea by increasing dendritic cell maturation and enhancing Th1 cytokine profiles (113), while *B. clausii* induced epithelial stabilization, reduced oxidative stress, and upregulated type I IFN pathways, curbing RV replication *in vitro* (114). In pigs, probiotics modulated T cell responses, increasing CD8 + T cells and IFN-*γ* levels, which are critical for viral clearance (115). Probiotics also reduced RV infection in porcine IPEC-J2 epithelial cells by enhancing expression of tight junction proteins (claudin-1, occludin), reducing viral adhesion, and decreasing NF-κB mediated inflammation (116–118).

**Table 7:** Modulation of RV infection by dietary fibers, prebiotics, probiotics, and synbiotics.

Category	Intervention/component	Experimental model	Main Findings	Mechanism	Reference
Dietary fiber	Rice bran	pigs	Reduced RV diarrhea; promoted Th1 immune responses to HRV vaccine	Enhanced gut barrier, increased probiotic growth, upregulated IFN-γ	(103, 104)
	Rice bran + probiotics	Pigs	Altered intestinal and serum metabolome; protection against HRV diarrhea	Modulated gut microbiota, increased SCFAs, improved innate immunity	(107)
Prebiotic	scGOS/lcFOS	Suckling rats	Reduced incidence/severity of RV diarrhea	Increased beneficial bacteria, enhanced mucosal immunity	(108)
	Prebiotics (unspecified)	Piglets	Decreased diarrhea and gut damage during RV infection	Modulated gut microbiota, reduced gut inflammation	(106)
	Prebiotics + bifidobacteria	Mice	Enhanced immune responses in RV-challenged mice	Increased mucosal IgA and cytokine production	(111)
	Prebiotics + postbiotics	Rats	Prevented RV-induced diarrhea	Enhanced gut barrier integrity, modulated microbiota	(112)
Probiotic	Various probiotics (e.g., *L. rhamnosus* GG, *B. breve*, *B. clausii*)	Pigs, rats, humans	Reduced RV infection, improved gut health, modulated immune responses	Strain-specific adherence, priming of innate immunity, increased IFN-γ, IL-12	(72, 113, 114, 126, 127)
	Lactic-acid bacteria	Mice	Primed innate immunity, protected against RV	Upregulated type I IFN, dendritic cell activation	(128)
	Probiotics + zinc	Indian infants	No consistent effect on oral RV vaccine response	Variable immunomodulatory impact	(125)
	Probiotics + zinc + selenium	Children	Improved recovery in RV enteritis	Synergistic immune enhancement	(87)
	Bifidobacteria and Lactobacilli	IPEC-J2 cells	Reduced porcine RV OSU infection	Blocked virus adhesion, maintained epithelial integrity	(116)
	*Bacillus clausii*	In vitro	Inhibited RV infection	Stabilized gut epithelium, modulated immune markers	(114)
	*Bifidobacterium longum* BORI and *Lactobacillus acidophilus*	Children	Reduced diarrhea duration and severity	Competitive exclusion, immunomodulation	(127, 129)
Synbiotic	scGOS/lcFOS + *B. breve* M-16 V	Suckling rats	Reduced incidence/severity of RV diarrhea	Enhanced gut microbiota composition, upregulated mucosal immunity	(108, 119)
	Probiotics + Prebiotics	Mice	Modulated RV gastroenteritis severity	Synergistic microbiota modulation, enhanced IgA production	(4)
	Fermented milk concentrate + prebiotics	Suckling rats	Prevented RV diarrhea	Enhanced gut barrier, immune modulation	(120)
	Lactic-acid bacteria + RV vaccine candidate	In vitro/Preclinical	Induced virus-specific immunity	Expression of viral proteins, adjuvant effect	(130)

Synbiotics, like scGOS/lcFOS combined with *B. breve* M-16 V, provided synergistic protection by reinforcing the epithelial barrier, elevating mucosal IgA, and stimulating TLR expression, leading to heightened pathogen recognition and immune activation (108, 119). A fermented milk with prebiotics induced similar protection via upregulating mucin production and fortifying tight junctions (20, 120). Additionally, a probiotic-prebiotic combination reduced severe gastroenteritis in mice by increasing intestinal IFN-*γ* and IL-10 levels, balancing inflammation (4). Together, these interventions work by a multifaceted mechanism involving gut microbiota modulation, enhancement of epithelial integrity, and immune system priming, leading to effective mitigation of RV infection. Incorporating dietary fibers, prebiotics, and probiotics into nutritional strategies offers a promising approach to bolster gut health, enhance immune defenses, and reduce the impact of RV infections.

## 5 Gaps in knowledge and future directions

**Figure 1:**
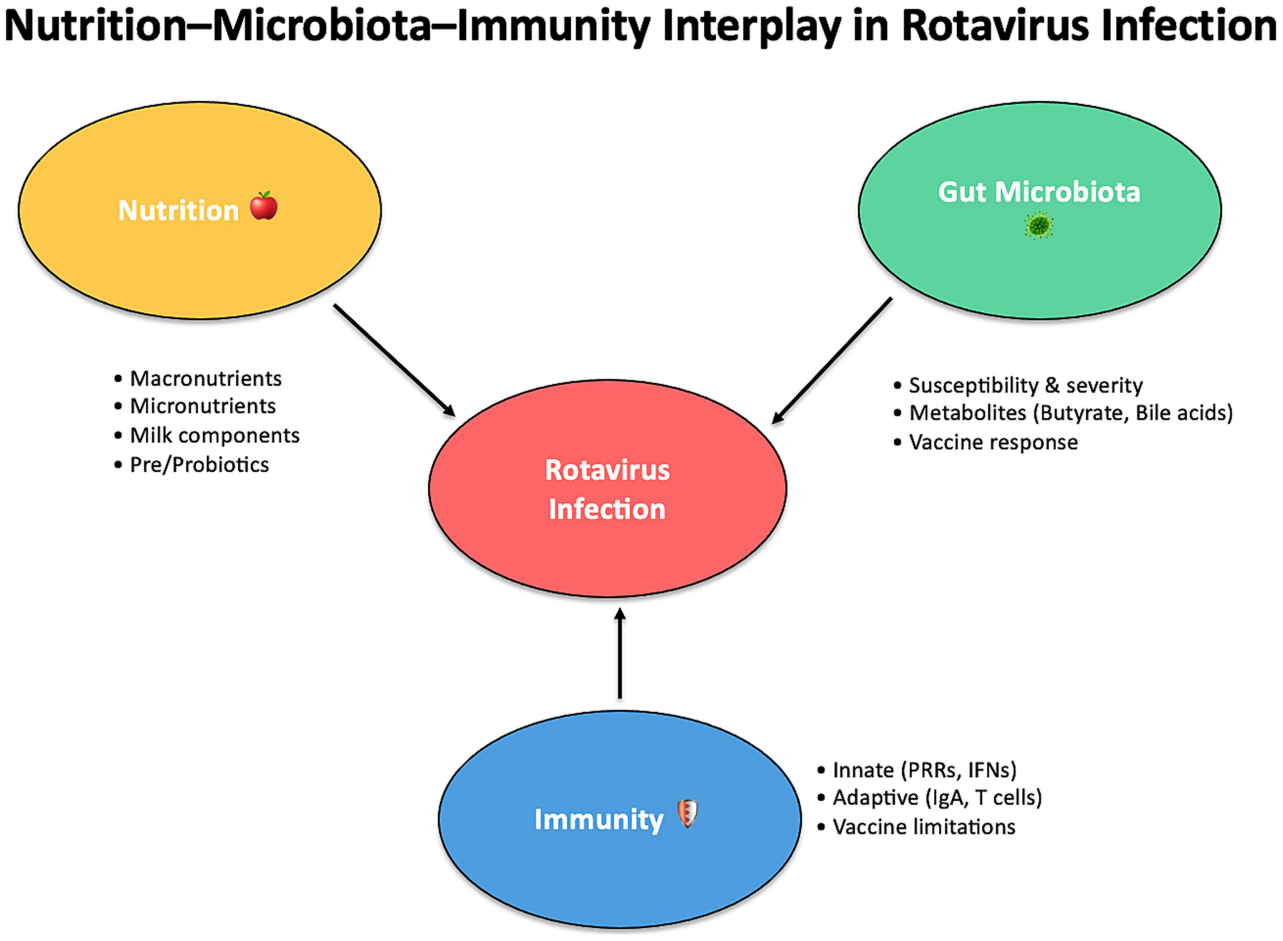
The interplay between nutrition, microbiota, and immunity in RV infection.

The interplay between nutrition, gut microbiota, and immunity is a cornerstone of the body’s response to RV infection, influencing both the susceptibility to infection and the severity of the disease. RV, as a leading cause of diarrheal disease, remains a major global health challenge, particularly in LMICswhere high rates of malnutrition and dysbiosis increase the vulnerability of populations to more severe outcomes (1). The recent research reviewed herein highlights the multifaceted roles that dietary factors, microbiota composition, and immune responses play in shaping the overall RV disease process. Nutrition exerts profound effects on the gut microbiota, which in turn influences immune responses critical for defending against RV infection. Macronutrients, micronutrients, probiotics, and prebiotics contribute to maintaining a balanced and diverse microbiota, which is essential for promoting efficient immune responses, strengthening gut barrier integrity, and reducing disease severity. The ability of specific dietary components to modulate immune function is a promising therapeutic avenue for improving RV outcomes, particularly when used in conjunction with vaccines (Figure 1).

Despite significant advancements in understanding the interplay between nutrition, microbiota, and immunity in RV infection, several critical knowledge gaps remain. Addressing these gaps is essential to develop more effective prevention and therapeutic strategies, particularly in vulnerable populations with high disease burdens. While numerous studies have established associations between gut microbiota composition and immune responses to RV, the specific molecular and cellular mechanisms underlying these interactions are not fully elucidated. Future research should focus on delineating how specific microbial taxa and their metabolites modulate mucosal and systemic immunity against RV. The variability in oral RV vaccine efficacy between high-income and LMICsremains poorly understood. Factors such as environmental enteric dysfunction, microbiota composition, nutritional deficiencies, and genetic diversity likely contribute to these differences. Comprehensive multi-omic studies integrating microbiome, metabolome, and host genetic data are needed to identify key determinants of vaccine performance. Although numerous nutritional interventions (e.g., probiotics, prebiotics, micronutrients) have shown promise in modulating gut health and immunity, the optimal combinations, dosages, and timing of these interventions remain to be established. More randomized controlled trials are necessary to define standardized, population-specific nutritional protocols. Personalized nutrition and microbiota modulation offer great potential but face practical challenges in implementation, particularly in resource-limited settings. Developing cost-effective, scalable methods for microbiota profiling and individualized dietary interventions is a critical area for future research. The long-term effects of early-life nutritional and microbiota-targeted interventions on RV immunity, susceptibility to other enteric infections, and overall health remain unclear. Longitudinal cohort studies are needed to assess how early interventions influence immune development and disease outcomes across the lifespan. Environmental factors such as sanitation, hygiene, and exposure to pathogens significantly influence gut microbiota and immunity. Future studies should integrate environmental and socioeconomic variables to develop holistic interventions that address the broader determinants of health affecting RV outcomes. Innovative vaccine strategies that harness or modulate the microbiota to enhance immune responses are still in nascent stages. Research into microbiota-directed vaccines and adjuvants could revolutionize vaccination strategies, especially in populations with dysbiosis or compromised gut health. Implementing personalized nutrition and microbiome-based interventions raises ethical considerations related to data privacy, equity in access, and informed consent. Recent progress in three-dimensional intestinal organoid technology has also provided robust and physiologically relevant platforms for investigating RV–host interactions. This technology complements existing human, animal, and *in vitro* models and holds great promise for advancing mechanistic understanding and therapeutic discovery in RV infection. Recent advances in artificial intelligence and machine learning offer promising avenues for dissecting the complex interplay between nutrition, microbiota, and immune responses in rotavirus infection. These applications in clinical environments are evolving—encompassing tasks ranging from diagnostic support to therapeutic stratification—and are poised to bridge the gap between bench findings and real-world implementation (121). In the context of our topic, these tools could be leveraged to integrate microbiome composition, nutritional status, immune profiles, and clinical outcomes, thereby refining intervention strategies. Establishing ethical frameworks and guidelines will be essential as these approaches move closer to clinical and public health applications. Future research should prioritize interdisciplinary collaborations integrating microbiology, immunology, nutrition, genomics, and public health. Investments in technological innovations, such as low-cost microbiota sequencing and machine learning algorithms for predictive modeling, will accelerate progress. Policymakers and healthcare providers must also be engaged to translate scientific insights into practical, equitable interventions that can be implemented globally. By addressing these knowledge gaps and advancing integrated strategies, the scientific and medical communities can enhance RV prevention, treatment, and vaccine efficacy, ultimately reducing the global burden of this significant enteric pathogen.

## References

[1] Franco MA Greenberg HB. Challenges for rotavirus vaccines. Virology. (2001) 281:153–5. doi: 10.1006/viro.2001.0830 11277688

[2] Magwira CA Taylor MB. Composition of gut microbiota and its influence on the immunogenicity of oral rotavirus vaccines. Vaccine. (2018) 36:3427–33. doi: 10.1016/j.vaccine.2018.04.091, PMID: 29752022

[3] Vlasova AN Paim FC Kandasamy S Alhamo MA Fischer DD Langel SN. Protein malnutrition modifies innate immunity and gene expression by intestinal epithelial cells and human rotavirus infection in neonatal gnotobiotic pigs. mSphere. (2017) 2:17. doi: 10.1128/mSphere.00046-17 PMC5332602 28261667

[4] Gonzalez-Ochoa G Flores-Mendoza LK Icedo-Garcia R Gomez-Flores R Tamez-Guerra P. Modulation of rotavirus severe gastroenteritis by the combination of probiotics and prebiotics. Arch Microbiol. (2017) 199:953–61. doi: 10.1007/s00203-017-1400-3, PMID: 28634691 PMC5548957

[5] Angel J Franco MA Greenberg HB. Rotavirus immune responses and correlates of protection. Curr Opin Virol. (2012) 2:419–25. doi: 10.1016/j.coviro.2012.05.003, PMID: 22677178 PMC3422408

[6] Holloway G Coulson BS. Innate cellular responses to rotavirus infection. J Gen Virol. (2013) 94:1151–60. doi: 10.1099/vir.0.051276-0, PMID: 23486667

[7] Li E Feng N Zeng Q Sanchez-Tacuba L Kawagishi T Branham G. Rhesus rotavirus NSP1 mediates extra-intestinal infection and is a contributing factor for biliary obstruction. PLoS Pathog. (2024) 20:e1012609. doi: 10.1371/journal.ppat.1012609, PMID: 39348381 PMC11476687

[8] Yuan L Saif LJ. Induction of mucosal immune responses and protection against enteric viruses: rotavirus infection of gnotobiotic pigs as a model. Vet Immunol Immunopathol. (2002) 87:147–60. doi: 10.1016/s0165-2427(02)00046-6, PMID: 12072229 PMC7119626

[9] Desselberger U Huppertz HI. Immune responses to rotavirus infection and vaccination and associated correlates of protection. J Infect Dis. (2011) 203:188–95. doi: 10.1093/infdis/jiq031, PMID: 21288818 PMC3071058

[10] Cohen AL Platts-Mills JA Nakamura T Operario DJ Antoni S Mwenda JM. Aetiology and incidence of diarrhoea requiring hospitalisation in children under 5 years of age in 28 low-income and middle-income countries: findings from the global Pediatric Diarrhea surveillance network. BMJ Glob Health. (2022) 7:548. doi: 10.1136/bmjgh-2022-009548, PMID: 36660904 PMC9445824

[11] Angel J Steele AD Franco MA. Correlates of protection for rotavirus vaccines: possible alternative trial endpoints, opportunities, and challenges. Hum Vaccin Immunother. (2014) 10:3659–71. doi: 10.4161/hv.34361, PMID: 25483685 PMC4514048

[12] Grimwood K Lambert SB. Rotavirus vaccines: opportunities and challenges. Hum Vaccin. (2009) 5:57–69. doi: 10.4161/hv.5.2.6924, PMID: 18838873

[13] Burnett E Parashar U Tate J. Rotavirus vaccines: effectiveness, safety, and future directions. Paediatr Drugs. (2018) 20:223–33. doi: 10.1007/s40272-018-0283-3, PMID: 29388076 PMC5955791

[14] Carvalho MF Gill D. Rotavirus vaccine efficacy: current status and areas for improvement. Hum Vaccin Immunother. (2019) 15:1237–50. doi: 10.1080/21645515.2018.1520583, PMID: 30215578 PMC6663136

[15] Heyman M Corthier G Petit A Meslin JC Moreau C Desjeux JF. Intestinal absorption of macromolecules during viral enteritis: an experimental study on rotavirus-infected conventional and germ-free mice. Pediatr Res. (1987) 22:72–8. doi: 10.1203/00006450-198707000-00017, PMID: 3627876 PMC7086805

[16] Hulst M Kerstens H de Wit A Smits M van der Meulen J Niewold T. Early transcriptional response in the jejunum of germ-free piglets after oral infection with virulent rotavirus. Arch Virol. (2008) 153:1311–22. doi: 10.1007/s00705-008-0118-6, PMID: 18523839 PMC2441536

[17] Uchiyama R Chassaing B Zhang B Gewirtz AT. Antibiotic treatment suppresses rotavirus infection and enhances specific humoral immunity. J Infect Dis. (2014) 210:171–82. doi: 10.1093/infdis/jiu037, PMID: 24436449 PMC4399425

[18] Ngo VL Wang Y Wang Y Shi Z Britton R Zou J. Select Gut Microbiota Impede Rotavirus Vaccine Efficacy. Cell Mol Gastroenterol Hepatol. (2024) 18:101393. doi: 10.1016/j.jcmgh.2024.101393, PMID: 39179176 PMC11462264

[19] Li E Li C Horn N Ajuwon KM. PPARgamma activation inhibits endocytosis of claudin-4 and protects against deoxynivalenol-induced intestinal barrier dysfunction in IPEC-J2 cells and weaned piglets. Toxicol Lett. (2023) 375:8–20. doi: 10.1016/j.toxlet.2022.12.015, PMID: 36596350

[20] Li E Li C Horn N Ajuwon KM. Quercetin attenuates deoxynivalenol-induced intestinal barrier dysfunction by activation of Nrf2 signaling pathway in IPEC-J2 cells and weaned piglets. Curr Res Toxicol. (2023) 5:100122. doi: 10.1016/j.crtox.2023.100122, PMID: 37720305 PMC10500468

[21] Chanez-Paredes SD Abtahi S Zha J Li E Marsischky G Zuo L. Mechanisms underlying distinct subcellular localization and regulation of epithelial long myosin light-chain kinase splice variants. J Biol Chem. (2024) 300:105643. doi: 10.1016/j.jbc.2024.105643, PMID: 38199574 PMC10862019

[22] Zhao Y Hu N Jiang Q Zhu L Zhang M Jiang J. Protective effects of sodium butyrate on rotavirus inducing endoplasmic reticulum stress-mediated apoptosis via PERK-eIF2alpha signaling pathway in IPEC-J2 cells. J Anim Sci Biotechnol. (2021) 12:69. doi: 10.1186/s40104-021-00592-0, PMID: 34112268 PMC8194137

[23] Dong X Wang Y Zhu X Shen L Chen L Niu L. Sodium butyrate protects against rotavirus-induced intestinal epithelial barrier damage by activating AMPK-Nrf2 signaling pathway in IPEC-J2 cells. Int J Biol Macromol. (2023) 228:186–96. doi: 10.1016/j.ijbiomac.2022.12.219, PMID: 36565836

[24] Li E Ajuwon KM. Mechanism of endocytic regulation of intestinal tight junction remodeling during nutrient starvation in jejunal IPEC-J2 cells. FASEB J. (2021) 35:e21356. doi: 10.1096/fj.202002098R, PMID: 33484473

[25] Li E Horn N Ajuwon KM. Mechanisms of deoxynivalenol-induced endocytosis and degradation of tight junction proteins in jejunal IPEC-J2 cells involve selective activation of the MAPK pathways. Arch Toxicol. (2021) 95:2065–79. doi: 10.1007/s00204-021-03044-w, PMID: 33847777

[26] Kim Y Chang KO. Inhibitory effects of bile acids and synthetic farnesoid X receptor agonists on rotavirus replication. J Virol. (2011) 85:12570–7. doi: 10.1128/JVI.05839-11, PMID: 21957312 PMC3209393

[27] Kong F Saif LJ Wang Q. Roles of bile acids in enteric virus replication. Anim Dis. (2021) 1:2. doi: 10.1186/s44149-021-00003-x, PMID: 34778876 PMC8062211

[28] Huang Y Zhu Q Wang Y Zhu K. Bacterial-derived sialidases inhibit porcine rotavirus OSU replication by interfering with the early steps of infection. Microb Pathog. (2024) 190:106628. doi: 10.1016/j.micpath.2024.106628, PMID: 38508422

[29] Bialowas S Hagbom M Nordgren J Karlsson T Sharma S Magnusson KE. Rotavirus and serotonin Cross-talk in diarrhoea. PLoS One. (2016) 11:e0159660. doi: 10.1371/journal.pone.0159660, PMID: 27459372 PMC4961431

[30] Mametja PM Motshudi MC Naidoo CM Rakau K Seheri LM Mkolo NM. Tapping into metabolomics for understanding host and rotavirus group a interactome. Life (Basel). (2025) 15:15. doi: 10.3390/life15050765, PMID: 40430193 PMC12113392

[31] Vorobiova N Usachova E. Influence of carbohydrate malabsorption syndrome on the clinical course of rotavirus infection in children at an early age. Georgian Med News. (2021) 311:120–5. PMID: 33814404

[32] Harris VC Haak BW Handley SA Jiang B Velasquez DE Hykes BL. Effect of antibiotic-mediated microbiome modulation on rotavirus vaccine immunogenicity: a human, randomized-control proof-of-concept trial. Cell Host Microbe. (2018) 24:197–207.e194. doi: 10.1016/j.chom.2018.07.005, PMID: 30092197 PMC11514417

[33] Majamaa H Isolauri E Saxelin M Vesikari T. Lactic acid bacteria in the treatment of acute rotavirus gastroenteritis. J Pediatr Gastroenterol Nutr. (1995) 20:333–8. doi: 10.1097/00005176-199504000-00012, PMID: 7608829

[34] Lee DK Park JE Kim MJ Seo JG Lee JH Ha NJP. Acidophilus inhibit infection by rotavirus in vitro and decrease the duration of diarrhea in pediatric patients. Clin Res Hepatol Gastroenterol. (2015) 39:237–44. doi: 10.1016/j.clinre.2014.09.006, PMID: 25459995

[35] Pant N Marcotte H Brussow H Svensson L Hammarstrom L. Effective prophylaxis against rotavirus diarrhea using a combination of *Lactobacillus rhamnosus* GG and antibodies. BMC Microbiol. (2007) 7:86. doi: 10.1186/1471-2180-7-86, PMID: 17900343 PMC2194776

[36] Kang JY Lee DK Ha NJ Shin HS. Antiviral effects of *Lactobacillus ruminis* SPM0211 and *Bifidobacterium longum* SPM1205 and SPM1206 on rotavirus-infected Caco-2 cells and a neonatal mouse model. J Microbiol. (2015) 53:796–803. doi: 10.1007/s12275-015-5302-2, PMID: 26502964 PMC7090939

[37] Shi Z Zou J Zhang Z Zhao X Noriega J Zhang B. Segmented filamentous Bacteria prevent and cure rotavirus infection. Cell. (2019) 179:644-658 e613. doi: 10.1016/j.cell.2019.09.028, PMID: 31607511 PMC7525827

[38] Ngo VL Shi Z Jiang B Gewirtz AT. Segmented filamentous bacteria impede rotavirus infection via retinoic acid receptor-mediated signaling. Gut Microbes. (2023) 15:2174407. doi: 10.1080/19490976.2023.2174407, PMID: 36740862 PMC9904313

[39] Varyukhina S Freitas M Bardin S Robillard E Tavan E Sapin C. Glycan-modifying bacteria-derived soluble factors from Bacteroides thetaiotaomicron and *Lactobacillus casei* inhibit rotavirus infection in human intestinal cells. Microbes Infect. (2012) 14:273–8. doi: 10.1016/j.micinf.2011.10.007, PMID: 22079149

[40] Seo BJ Mun MR J R K Kim CJ Lee I Chang YH. Bile tolerant *Lactobacillus reuteri* isolated from pig feces inhibits enteric bacterial pathogens and porcine rotavirus. Vet Res Commun. (2010) 34:323–33. doi: 10.1007/s11259-010-9357-6, PMID: 20396947

[41] Juntunen M Kirjavainen PV Ouwehand AC Salminen SJ Isolauri E. Adherence of probiotic bacteria to human intestinal mucus in healthy infants and during rotavirus infection. Clin Diagn Lab Immunol. (2001) 8:293–6. doi: 10.1128/CDLI.8.2.293-296.2001, PMID: 11238211 PMC96052

[42] Zhang W Wen K Azevedo MS Gonzalez A Saif LJ Li G. Lactic acid bacterial colonization and human rotavirus infection influence distribution and frequencies of monocytes/macrophages and dendritic cells in neonatal gnotobiotic pigs. Vet Immunol Immunopathol. (2008) 121:222–31. doi: 10.1016/j.vetimm.2007.10.001, PMID: 18006076 PMC2268605

[43] Kgosana LP Seheri ML Magwira CA. Abundance of selected lipopolysaccharide-Rich Bacteria and levels of toll-like receptor 4 and interleukin 8 expression are significantly associated with live attenuated rotavirus vaccine shedding among south African infants. Vaccines (Basel). (2024) 12:273. doi: 10.3390/vaccines12030273, PMID: 38543907 PMC10974388

[44] Raev SA Omwando AM Guo Y Raque MS Amimo JO Saif LJ. Glycan-mediated interactions between bacteria, rotavirus and the host cells provide an additional mechanism of antiviral defence. Benef Microbes. (2022) 13:383–96. doi: 10.3920/BM2022.0026, PMID: 36239669

[45] Engevik MA Banks LD Engevik KA Chang-Graham AL Perry JL Hutchinson DS. Rotavirus infection induces glycan availability to promote ileum-specific changes in the microbiome aiding rotavirus virulence. Gut Microbes. (2020) 11:1324–47. doi: 10.1080/19490976.2020.1754714, PMID: 32404017 PMC7524290

[46] Parker EPK Praharaj I Zekavati A Lazarus RP Giri S Operario DJ. Influence of the intestinal microbiota on the immunogenicity of oral rotavirus vaccine given to infants in South India. Vaccine. (2018) 36:264–72. doi: 10.1016/j.vaccine.2017.11.031, PMID: 29217369 PMC5755003

[47] Saavedra JM Bauman NA Oung I Perman JA Yolken RH. Feeding of Bifidobacterium bifidum and *Streptococcus thermophilus* to infants in hospital for prevention of diarrhoea and shedding of rotavirus. Lancet. (1994) 344:1046–9. doi: 10.1016/s0140-6736(94)91708-6, PMID: 7934445

[48] Fischer DD Kandasamy S Paim FC Langel SN Alhamo MA Shao L. Protein malnutrition alters tryptophan and angiotensin-converting enzyme 2 homeostasis and adaptive immune responses in human rotavirus-infected gnotobiotic pigs with human infant Fecal microbiota transplant. Clin Vaccine Immunol. (2017) 24:17. doi: 10.1128/CVI.00172-17, PMID: 28637803 PMC5583476

[49] Zijlstra RT Donovan SM Odle J Gelberg HB Petschow BW Gaskins HR. Protein-energy malnutrition delays small-intestinal recovery in neonatal pigs infected with rotavirus. J Nutr. (1997) 127:1118–27. doi: 10.1093/jn/127.6.1118, PMID: 9187626

[50] Zijlstra RT McCracken BA Odle J Donovan SM Gelberg HB Petschow BW. Malnutrition modifies pig small intestinal inflammatory responses to rotavirus. J Nutr. (1999) 129:838–43. doi: 10.1093/jn/129.4.838, PMID: 10203558

[51] Oladele P Li E Lu H Cozannet P Nakatsu C Johnson T. Effect of a carbohydrase admixture in growing pigs fed wheat-based diets in thermoneutral and heat stress conditions. J Anim Sci. (2021) 99:254. doi: 10.1093/jas/skab254, PMID: 34460910 PMC8562353

[52] Kumar A Vlasova AN Deblais L Huang HC Wijeratne A Kandasamy S. Impact of nutrition and rotavirus infection on the infant gut microbiota in a humanized pig model. BMC Gastroenterol. (2018) 18:93. doi: 10.1186/s12876-018-0810-2, PMID: 29929472 PMC6013989

[53] Srivastava V Deblais L Huang HC Miyazaki A Kandasamy S Langel SN. Reduced rotavirus vaccine efficacy in protein malnourished human-faecal-microbiota-transplanted gnotobiotic pig model is in part attributed to the gut microbiota. Benef Microbes. (2020) 11:733–51. doi: 10.3920/BM2019.0139 33245014

[54] Perez-Cano FJ Marin-Gallen S Castell M Rodriguez-Palmero M Rivero M Castellote C. Supplementing suckling rats with whey protein concentrate modulates the immune response and ameliorates rat rotavirus-induced diarrhea. J Nutr. (2008) 138:2392–8. doi: 10.3945/jn.108.093856, PMID: 19022963

[55] Corl BA Harrell RJ Moon HK Phillips O Weaver EM Campbell JM. Effect of animal plasma proteins on intestinal damage and recovery of neonatal pigs infected with rotavirus. J Nutr Biochem. (2007) 18:778–84. doi: 10.1016/j.jnutbio.2006.12.011, PMID: 17475463

[56] Wolber FM Broomfield AM Fray L Cross ML Dey D. Supplemental dietary whey protein concentrate reduces rotavirus-induced disease symptoms in suckling mice. J Nutr. (2005) 135:1470–4. doi: 10.1093/jn/135.6.1470, PMID: 15930454

[57] Wang W Chen Y Wang J Lv Z Li E Zhao J. Effects of reduced dietary protein at high temperature in summer on growth performance and carcass quality of finishing pigs. Animals (Basel). (2022) 12:599. doi: 10.3390/ani12050599, PMID: 35268168 PMC8909873

[58] Fu Y Li E Casey TM Johnson TA Adeola O Ajuwon KM. Impact of maternal live yeast supplementation to sows on intestinal inflammatory cytokine expression and tight junction proteins in suckling and weanling piglets. J Anim Sci. (2024) 102:8. doi: 10.1093/jas/skae008, PMID: 38206189 PMC10836509

[59] Monaco MH Gross G Donovan SM. Whey protein lipid concentrate high in Milk fat globule membrane components inhibit porcine and human rotavirus in vitro. Front Pediatr. (2021) 9:731005. doi: 10.3389/fped.2021.731005, PMID: 34540774 PMC8442734

[60] Superti F Marziano ML Donelli G Marchetti M Seganti L. Enhancement of rotavirus infectivity by saturated fatty acids. Comp Immunol Microbiol Infect Dis. (1995) 18:129–35. doi: 10.1016/0147-9571(95)98854-b, PMID: 7621669

[61] Tao R Cheng X Gu L Zhou J Zhu X Zhang X. Lipidomics reveals the significance and mechanism of the cellular ceramide metabolism for rotavirus replication. J Virol. (2024) 98:e0006424. doi: 10.1128/jvi.00064-24, PMID: 38488360 PMC11019908

[62] Mirazimi A Svensson L. Carbohydrates facilitate correct disulfide bond formation and folding of rotavirus VP7. J Virol. (1998) 72:3887–92. doi: 10.1128/JVI.72.5.3887-3892.1998, PMID: 9557673 PMC109613

[63] Sack DA Rhoads M Molla A Molla AM Wahed MA. Carbohydrate malabsorption in infants with rotavirus diarrhea. Am J Clin Nutr. (1982) 36:1112–8. doi: 10.1093/ajcn/36.6.1112, PMID: 7148733

[64] Isa P Arias CF Lopez S. Role of sialic acids in rotavirus infection. Glycoconj J. (2006) 23:27–37. doi: 10.1007/s10719-006-5435-y, PMID: 16575520 PMC7087688

[65] Srnka CA Tiemeyer M Gilbert JH Moreland M Schweingruber H de Lappe BW. Cell surface ligands for rotavirus: mouse intestinal glycolipids and synthetic carbohydrate analogs. Virology. (1992) 190:794–805. doi: 10.1016/0042-6822(92)90917-e, PMID: 1325706

[66] Zhou X Li Y Li T Cao J Guan Z Xu T. Polysaccharide inhibits porcine rotavirus in vitro. Animals (Basel). (2023) 13:306. doi: 10.3390/ani13142306, PMID: 37508085 PMC10376577

[67] Li Y Zhou X Qi S Jia G Cao J Guan Z. Polysaccharide on piglets infected with porcine rotavirus. Microb Pathog. (2025) 200:107355. doi: 10.1016/j.micpath.2025.107355, PMID: 39892834

[68] Ahmed F Jones DB Jackson AA. The interaction of vitamin a deficiency and rotavirus infection in the mouse. Br J Nutr. (1990) 63:363–73. doi: 10.1079/bjn19900122, PMID: 2334670

[69] Reifen R Mor A Nyska A. Vitamin a deficiency aggravates rotavirus infection in CD-1 mice through extensive involvement of the gut. Int J Vitam Nutr Res. (2004) 74:355–61. doi: 10.1024/0300-9831.74.5.355, PMID: 15628674

[70] Ahmed F Jones DB Jackson AA. Effect of vitamin a deficiency on the immune response to epizootic diarrhoea of infant mice (EDIM) rotavirus infection in mice. Br J Nutr. (1991) 65:475–85. doi: 10.1079/bjn19910106, PMID: 1652282

[71] Vlasova AN Chattha KS Kandasamy S Siegismund CS Saif LJ. Prenatally acquired vitamin a deficiency alters innate immune responses to human rotavirus in a gnotobiotic pig model. J Immunol. (2013) 190:4742–53. doi: 10.4049/jimmunol.1203575, PMID: 23536630 PMC3633669

[72] Chattha KS Kandasamy S Vlasova AN Saif LJ. Vitamin a deficiency impairs adaptive B and T cell responses to a prototype monovalent attenuated human rotavirus vaccine and virulent human rotavirus challenge in a gnotobiotic piglet model. PLoS One. (2013) 8:e82966. doi: 10.1371/journal.pone.0082966, PMID: 24312675 PMC3846786

[73] Kandasamy S Chattha KS Vlasova AN Saif LJ. Prenatal vitamin A deficiency impairs adaptive immune responses to pentavalent rotavirus vaccine (RotaTeq(R)) in a neonatal gnotobiotic pig model. Vaccine. (2014) 32:816–24. doi: 10.1016/j.vaccine.2013.12.039, PMID: 24380684

[74] Chepngeno J Amimo JO Michael H Jung K Raev S Lee MV. Rotavirus a inoculation and Oral vitamin a supplementation of vitamin a deficient pregnant sows enhances maternal adaptive immunity and passive protection of piglets against virulent rotavirus a. Viruses. (2022) 14:12354. doi: 10.3390/v14112354, PMID: 36366453 PMC9697517

[75] Chepngeno J Amimo JO Michael H Raev SA Jung K Lee MV. Vitamin a deficiency and vitamin a supplementation affect innate and T cell immune responses to rotavirus a infection in a conventional sow model. Front Immunol. (2023) 14:1188757. doi: 10.3389/fimmu.2023.1188757, PMID: 37180172 PMC10166828

[76] Tian G Liang X Chen D Mao X Yu J Zheng P. Vitamin D3 supplementation alleviates rotavirus infection in pigs and IPEC-J2 cells via regulating the autophagy signaling pathway. J Steroid Biochem Mol Biol. (2016) 163:157–63. doi: 10.1016/j.jsbmb.2016.05.004, PMID: 27174720

[77] Bucak IH Ozturk AB Almis H Cevik MO Tekin M Konca C. Is there a relationship between low vitamin D and rotaviral diarrhea? Pediatr Int. (2016) 58:270–3. doi: 10.1111/ped.12809, PMID: 26287796

[78] Zhao Y Yu B Mao X He J Huang Z Zheng P. Effect of 25-hydroxyvitamin D3 on rotavirus replication and gene expressions of RIG-I signalling molecule in porcine rotavirus-infected IPEC-J2 cells. Arch Anim Nutr. (2015) 69:227–35. doi: 10.1080/1745039X.2015.1034522, PMID: 25897656

[79] Zhao Y Ran Z Jiang Q Hu N Yu B Zhu L. Vitamin D alleviates rotavirus infection through a Microrna-155-5p mediated regulation of the TBK1/IRF3 Signaling pathway in vivo and in vitro. Int J Mol Sci. (2019) 20:3562. doi: 10.3390/ijms20143562, PMID: 31330869 PMC6678911

[80] Zhao Y Zhu X Lan Q Wei Z Shang P Song L. 1alpha,25-hydroxyvitamin D(3) alleviated rotavirus infection induced ferroptosis in IPEC-J2 cells by regulating the ATF3-SLC7A11-GPX4 axis. Int J Biol Macromol. (2024) 283:137484. doi: 10.1016/j.ijbiomac.2024.137484, PMID: 39528192

[81] Zhao Y Yu B Mao X He J Huang Z Zheng P. Dietary vitamin D supplementation attenuates immune responses of pigs challenged with rotavirus potentially through the retinoic acid-inducible gene I signalling pathway. Br J Nutr. (2014) 112:381–9. doi: 10.1017/S000711451400097X, PMID: 24833277

[82] Colgate ER Haque R Dickson DM Carmolli MP Mychaleckyj JC Nayak U. Delayed dosing of Oral rotavirus vaccine demonstrates decreased risk of rotavirus gastroenteritis associated with serum zinc: a randomized controlled trial. Clin Infect Dis. (2016) 63:634–41. doi: 10.1093/cid/ciw346, PMID: 27217217

[83] Dalgic N Sancar M Bayraktar B Pullu M Hasim O. Probiotic, zinc and lactose-free formula in children with rotavirus diarrhea: are they effective? Pediatr Int. (2011) 53:677–82. doi: 10.1111/j.1442-200X.2011.03325.x, PMID: 21261786

[84] Xu N Zhang W Huo J Tao R Jin T Zhang Y. Characterization of changes in the intestinal microbiome following combination therapy with zinc preparation and conventional treatment for children with rotavirus enteritis. Front Cell Infect Microbiol. (2023) 13:1153701. doi: 10.3389/fcimb.2023.1153701, PMID: 37842003 PMC10570505

[85] Erk I Huet JC Duarte M Duquerroy S Rey F Cohen J. A zinc ion controls assembly and stability of the major capsid protein of rotavirus. J Virol. (2003) 77:3595–601. doi: 10.1128/jvi.77.6.3595-3601.2003, PMID: 12610135 PMC149495

[86] Minaeian S Khales P Hosseini-Hosseinabad SM Farahmand M Poortahmasebi V Habib Z. Evaluation of activity of zinc oxide nanoparticles on human rotavirus and multi-drug resistant *Acinetobacter baumannii*. Pharm Nanotechnol. (2023) 11:475–85. doi: 10.2174/2211738511666230504121506, PMID: 37150981

[87] Cai Y Wang X Li C Li F Yan Z Ma N. Probiotics combined with zinc and selenium preparation in the treatment of child rotavirus enteritis. Am J Transl Res. (2022) 14:1043–50. PMID: 35273706 PMC8902533

[88] Hossieni M Kiani SJ Tavakoli A Kachooei A Habib Z Monavari SH. In vitro inhibition of rotavirus multiplication by copper oxide nanoparticles. Arch Razi Inst. (2024) 79:83–91. doi: 10.32592/ARI.2024.79.1.83, PMID: 39192955 PMC11345465

[89] Pando V Isa P Arias CF Lopez S. Influence of calcium on the early steps of rotavirus infection. Virology. (2002) 295:190–200. doi: 10.1006/viro.2001.1337, PMID: 12033777

[90] Cohen J Laporte J Charpilienne A Scherrer R. Activation of rotavirus RNA polymerase by calcium chelation. Arch Virol. (1979) 60:177–86. doi: 10.1007/BF01317489, PMID: 41504

[91] Poruchynsky MS Maass DR Atkinson PH. Calcium depletion blocks the maturation of rotavirus by altering the oligomerization of virus-encoded proteins in the ER. J Cell Biol. (1991) 114:651–6. doi: 10.1083/jcb.114.4.651, PMID: 1651336 PMC2289885

[92] Hester SN Chen X Li M Monaco MH Comstock SS Kuhlenschmidt TB. Human milk oligosaccharides inhibit rotavirus infectivity in vitro and in acutely infected piglets. Br J Nutr. (2013) 110:1233–42. doi: 10.1017/S0007114513000391, PMID: 23442265

[93] Laucirica DR Triantis V Schoemaker R Estes MK Ramani S. Milk oligosaccharides inhibit human rotavirus infectivity in MA104 cells. J Nutr. (2017) 147:1709–14. doi: 10.3945/jn.116.246090, PMID: 28637685 PMC5572490

[94] Li M Monaco MH Wang M Comstock SS Kuhlenschmidt TB Fahey GC. Human milk oligosaccharides shorten rotavirus-induced diarrhea and modulate piglet mucosal immunity and colonic microbiota. ISME J. (2014) 8:1609–20. doi: 10.1038/ismej.2014.10, PMID: 24522264 PMC4817601

[95] Ramani S Stewart CJ Laucirica DR Ajami NJ Robertson B Autran CA. Human milk oligosaccharides, milk microbiome and infant gut microbiome modulate neonatal rotavirus infection. Nat Commun. (2018) 9:5010. doi: 10.1038/s41467-018-07476-4, PMID: 30479342 PMC6258677

[96] Yolken RH Peterson JA Vonderfecht SL Fouts ET Midthun K Newburg DS. Human milk mucin inhibits rotavirus replication and prevents experimental gastroenteritis. J Clin Invest. (1992) 90:1984–91. doi: 10.1172/JCI116078, PMID: 1331178 PMC443262

[97] Newburg DS Peterson JA Ruiz-Palacios GM Matson DO Morrow AL Shults J. Role of human-milk lactadherin in protection against symptomatic rotavirus infection. Lancet. (1998) 351:1160–4. doi: 10.1016/s0140-6736(97)10322-1, PMID: 9643686

[98] Civra A Francese R Donalisio M Tonetto P Coscia A Sottemano S. Human colostrum and derived extracellular vesicles prevent infection by human rotavirus and respiratory syncytial virus in vitro. J Hum Lact. (2021) 37:122–34. doi: 10.1177/0890334420988239, PMID: 33534629

[99] Asensi MT Martinez-Costa C Buesa J. Anti-rotavirus antibodies in human milk: quantification and neutralizing activity. J Pediatr Gastroenterol Nutr. (2006) 42:560–7. doi: 10.1097/01.mpg.0000221892.59371.b3, PMID: 16707981

[100] Yu Y Lasanajak Y Song X Hu L Ramani S Mickum ML. Human milk contains novel glycans that are potential decoy receptors for neonatal rotaviruses. Mol Cell Proteomics. (2014) 13:2944–60. doi: 10.1074/mcp.M114.039875, PMID: 25048705 PMC4223483

[101] Kvistgaard AS Pallesen LT Arias CF Lopez S Petersen TE Heegaard CW. Inhibitory effects of human and bovine milk constituents on rotavirus infections. J Dairy Sci. (2004) 87:4088–96. doi: 10.3168/jds.S0022-0302(04)73551-1, PMID: 15545370

[102] Kazimbaya KM Chisenga CC Simuyandi M Phiri CM Laban NM Bosomprah S. In-vitro inhibitory effect of maternal breastmilk components on rotavirus vaccine replication and association with infant seroconversion to live oral rotavirus vaccine. PLoS One. (2020) 15:e0240714. doi: 10.1371/journal.pone.0240714, PMID: 33170860 PMC7654788

[103] Yang X Wen K Tin C Li G Wang H Kocher J. Dietary rice bran protects against rotavirus diarrhea and promotes Th1-type immune responses to human rotavirus vaccine in gnotobiotic pigs. Clin Vaccine Immunol. (2014) 21:1396–403. doi: 10.1128/CVI.00210-14, PMID: 25080551 PMC4266357

[104] Yang X Twitchell E Li G Wen K Weiss M Kocher J. High protective efficacy of rice bran against human rotavirus diarrhea via enhancing probiotic growth, gut barrier function, and innate immunity. Sci Rep. (2015) 5:15004. doi: 10.1038/srep15004, PMID: 26459937 PMC4602212

[105] Yang C Lu H Li E Oladele P Ajuwon KM. Inulin supplementation induces expression of hypothalamic antioxidant defence genes in weaned piglets. J Anim Physiol Anim Nutr (Berl). (2023) 107:157–64. doi: 10.1111/jpn.13698, PMID: 35253266

[106] Yang H Fan X Mao X Yu B He J Yan H. The protective role of prebiotics and probiotics on diarrhea and gut damage in the rotavirus-infected piglets. J Anim Sci Biotechnol. (2024) 15:61. doi: 10.1186/s40104-024-01018-3, PMID: 38698473 PMC11067158

[107] Nealon NJ Yuan L Yang X Ryan EP. Rice bran and probiotics Alter the porcine large intestine and serum metabolomes for protection against human rotavirus Diarrhea. Front Microbiol. (2017) 8:653. doi: 10.3389/fmicb.2017.00653, PMID: 28484432 PMC5399067

[108] Rigo-Adrover M Saldana-Ruiz S van Limpt K Knipping K Garssen J Knol J. A combination of scGOS/lcFOS with *Bifidobacterium breve* M-16V protects suckling rats from rotavirus gastroenteritis. Eur J Nutr. (2017) 56:1657–70. doi: 10.1007/s00394-016-1213-1, PMID: 27112962

[109] Wang J Tang L Wang Y Xing Y Chen G Jiang Q. Effects of enzymatic hydrolysate of cottonseed protein on growth performance, nutrient digestibility, blood indexes and Fecal volatile fatty acids of weaned piglets. J Anim Physiol Anim Nutr (Berl). (2025) 109:1062–71. doi: 10.1111/jpn.14121, PMID: 40222046

[110] Wang Y Li Z Chen G Xing Y Wang J Zhao Y. Dietary Galacto-oligosaccharides enhance growth performance and modulate gut microbiota in weaned piglets: a sustainable alternative to antibiotics. Animals (Basel). (2025) 15:508. doi: 10.3390/ani15111508, PMID: 40508974 PMC12153697

[111] Qiao H Duffy LC Griffiths E Dryja D Leavens A Rossman J. Immune responses in rhesus rotavirus-challenged BALB/c mice treated with bifidobacteria and prebiotic supplements. Pediatr Res. (2002) 51:750–5. doi: 10.1203/00006450-200206000-00015, PMID: 12032272

[112] Morales-Ferre C Azagra-Boronat I Massot-Cladera M Tims S Knipping K Garssen J. Preventive effect of a postbiotic and prebiotic mixture in a rat model of early life rotavirus induced-Diarrhea. Nutrients. (2022) 14:1163. doi: 10.3390/nu14061163, PMID: 35334820 PMC8954028

[113] Azagra-Boronat I Massot-Cladera M Knipping K Garssen J Ben Amor K Knol J. Strain-specific probiotic properties of bifidobacteria and lactobacilli for the prevention of Diarrhea caused by rotavirus in a preclinical model. Nutrients. (2020) 12:498. doi: 10.3390/nu12020498, PMID: 32075234 PMC7071190

[114] Paparo L Tripodi L Bruno C Pisapia L Damiano C Pastore L. Protective action of *Bacillus clausii* probiotic strains in an in vitro model of rotavirus infection. Sci Rep. (2020) 10:12636. doi: 10.1038/s41598-020-69533-7, PMID: 32724066 PMC7387476

[115] Chattha KS Vlasova AN Kandasamy S Rajashekara G Saif LJ. Divergent immunomodulating effects of probiotics on T cell responses to oral attenuated human rotavirus vaccine and virulent human rotavirus infection in a neonatal gnotobiotic piglet disease model. J Immunol. (2013) 191:2446–56. doi: 10.4049/jimmunol.1300678, PMID: 23918983 PMC4136549

[116] Leblanc D Raymond Y Lemay MJ Champagne CP Brassard J. Effect of probiotic bacteria on porcine rotavirus OSU infection of porcine intestinal epithelial IPEC-J2 cells. Arch Virol. (2022) 167:1999–2010. doi: 10.1007/s00705-022-05510-x, PMID: 35794494 PMC9402510

[117] Liu F Li G Wen K Bui T Cao D Zhang Y. Porcine small intestinal epithelial cell line (IPEC-J2) of rotavirus infection as a new model for the study of innate immune responses to rotaviruses and probiotics. Viral Immunol. (2010) 23:135–49. doi: 10.1089/vim.2009.0088, PMID: 20373994 PMC2883522

[118] Li E Horn N Ajuwon KM. EPA and DHA inhibit endocytosis of claudin-4 and protect against deoxynivalenol-induced intestinal barrier dysfunction through PPARgamma dependent and independent pathways in jejunal IPEC-J2 cells. Food Res Int. (2022) 157:111420. doi: 10.1016/j.foodres.2022.111420, PMID: 35761666

[119] Rigo-Adrover MDM van Limpt K Knipping K Garssen J Knol J Costabile A. Preventive effect of a Synbiotic combination of Galacto- and Fructooligosaccharides mixture with *Bifidobacterium breve* M-16V in a model of multiple rotavirus infections. Front Immunol. (2018) 9:1318. doi: 10.3389/fimmu.2018.01318, PMID: 29942312 PMC6004411

[120] Rigo-Adrover MDM Knipping K Garssen J van Limpt K Knol J Franch A. Prevention of rotavirus Diarrhea in suckling rats by a specific fermented Milk concentrate with prebiotic mixture. Nutrients. (2019) 11:189. doi: 10.3390/nu11010189, PMID: 30669251 PMC6356616

[121] Yuan H Yu K Xie F Liu M Sun S. Automated machine learning with interpretation: a systematic review of methodologies and applications in healthcare. Med Adv. (2024) 2:205–37. doi: 10.1002/med4.75

[122] Maier EA Weage KJ Guedes MM Denson LA McNeal MM Bernstein DI. Protein-energy malnutrition alters IgA responses to rotavirus vaccination and infection but does not impair vaccine efficacy in mice. Vaccine. (2013) 32:48–53. doi: 10.1016/j.vaccine.2013.10.072, PMID: 24200975 PMC3887447

[123] Miyazaki A Kandasamy S Michael H Langel SN Paim FC Chepngeno J. Protein deficiency reduces efficacy of oral attenuated human rotavirus vaccine in a human infant fecal microbiota transplanted gnotobiotic pig model. Vaccine. (2018) 36:6270–81. doi: 10.1016/j.vaccine.2018.09.008, PMID: 30219368 PMC6180620

[124] Ahmed F Jones DB Jackson AA. Effect of undernutrition on the immune response to rotavirus infection in mice. Ann Nutr Metab. (1990) 34:21–31. doi: 10.1159/000177566, PMID: 2331137

[125] Lazarus RP John J Shanmugasundaram E Rajan AK Thiagarajan S Giri S. The effect of probiotics and zinc supplementation on the immune response to oral rotavirus vaccine: a randomized, factorial design, placebo-controlled study among Indian infants. Vaccine. (2018) 36:273–9. doi: 10.1016/j.vaccine.2017.07.116, PMID: 28874323 PMC12001858

[126] Grandy G Medina M Soria R Teran CG Araya M. Probiotics in the treatment of acute rotavirus diarrhoea. A randomized, double-blind, controlled trial using two different probiotic preparations in Bolivian children. BMC Infect Dis. (2010) 10:253. doi: 10.1186/1471-2334-10-253, PMID: 20735858 PMC2940902

[127] Park MS Kwon B Ku S Ji GE. The efficacy of *Bifidobacterium longum* BORI and *Lactobacillus acidophilus* AD031 probiotic treatment in infants with rotavirus infection. Nutrients. (2017) 9:887. doi: 10.3390/nu9080887, PMID: 28813007 PMC5579680

[128] Thompson A Van Moorlehem E Aich P. Probiotic-induced priming of innate immunity to protect against rotaviral infection. Probiotics Antimicrob Proteins. (2010) 2:90–7. doi: 10.1007/s12602-009-9032-9, PMID: 26781117

[129] Huang YF Liu PY Chen YY Nong BR Huang IF Hsieh KS. Three-combination probiotics therapy in children with salmonella and rotavirus gastroenteritis. J Clin Gastroenterol. (2014) 48:37–42. doi: 10.1097/MCG.0b013e31828f1c6e, PMID: 23632352

[130] Afchangi A Latifi T Jalilvand S Marashi SM Shoja Z. Combined use of lactic-acid-producing bacteria as probiotics and rotavirus vaccine candidates expressing virus-specific proteins. Arch Virol. (2021) 166:995–1006. doi: 10.1007/s00705-021-04964-9, PMID: 33533975

